# Recreational screen time and mental health among Canadian children and youth

**DOI:** 10.24095/hpcdp.45.7/8.01

**Published:** 2025-08

**Authors:** Stephanie Toigo, Chinchin Wang, Stephanie A. Prince, Melanie Varin, Karen C. Roberts, Marisol T. Betancourt

**Affiliations:** 1 Centre for Surveillance and Applied Research, Public Health Agency of Canada, Ottawa, Ontario, Canada; 2 School of Epidemiology and Public Health, Faculty of Medicine, University of Ottawa, Ottawa, Ontario, Canada

**Keywords:** recreational screen time, mental health, children, youth, anxiety, child functioning, mood disorder

## Abstract

**Background:**

Higher amounts of recreational screen time have been associated with mental ill-health among children and youth. We examined the association between meeting the 24-Hour Movement Guideline’s recreational screen time recommendation of ≤2 hours/day and indicators of mental health among children and youth.

**Methods:**

Using the 2019 Canadian Health Survey on Children and Youth (N=26986), associations were assessed using age- and sex-stratified multivariate logistic regression. A secondary analysis used incremental amounts of screen time to explore dose–response relationships.

**Results:**

Female children (5–11 years) who met the recommendation were less likely to be diagnosed with an anxiety disorder (adjusted odds ratio=0.49; 99% CI: 0.25–0.96) or appear sad/depressed (0.60; 0.37–0.99). Female youth (12–17 years) who met the recommendation were more likely to report excellent or very good mental health, high happiness and high life satisfaction and less likely to report feeling stressed, anxious or depressed or be diagnosed with an anxiety disorder. Male youth who met the recommendation were more likely to report high happiness (1.74; 1.40–2.15) and high life satisfaction (1.64; 1.34–2.01) and less likely to feel stressed (0.74; 0.56–0.99) or experience psychosocial difficulties (0.79; 0.64–0.97). Some dose–response relationships were present among youth.

**Conclusion:**

Adherence to the screen time recommendation was associated with several mental health indicators. Understanding these associations can help inform future research and guide strategies to improve mental health.

**Highlights**

- Youth who met the recreational screen time recommendation of 2 hours or less per day were more likely than those who exceeded it to self-report high levels of positive mental health indicators, that is, excellent or very good mental health, high happiness and high life satisfaction.
- Youth who met the recommendation were less likely to report indicators of mental ill-health, such as often feeling stressed and psychosocial difficulties.
- The likelihood of youth reporting positive mental health indicators decreased as screen time increased.
- Female children, and not male children, who met the recommendation were less likely to often appear sad or depressed or be diagnosed with an anxiety disorder

## Introduction

Reducing sedentary screen time and increasing physical activity are public health priorities.^1^ Among children and youth, high levels of sedentary behaviour, including recreational screen time, are a risk factor for overweight and obesity, lower physical fitness and decreased self-esteem and prosocial behaviour.^2^

The Canadian 24-Hour Movement Guidelines recommend that children and youth aged 5 to 17 years accumulate no more than 2hours per day of recreational screen time.^3^-^5^ In 2018 to 2019, about 70% of Canadian children aged 5 to 11 years and 30% of youth aged 12 to 17 years met this recommendation.^1^ Recreational screen time use, among youth in particular, has increased since about 2000,^6^,^7^ and especially during the COVID-19 pandemic when the proportion of youth meeting the recreational screen time recommendation decreased from 33% in 2018 to 22% in 2021.^8^,^9^

Studies have found associations between sedentary screen time use and mental ill-health, where children and youth with higher screen time use reported more symptoms of depression, anxiety, hyperactivity and inattention.^10^-^13^ Sedentary screen time was also associated with greater severity of depression and anxiety symptoms among Canadian youth.^14^,^15^ Although the relationship between screen time and positive mental health has been less researched, a recent scoping review suggests that less sedentary behaviour is associated with greater mental wellness among children and youth.^16^

There has been little research examining associations between adherence to screen time recommendations and positive mental health indicators, mental ill-health, psychosocial difficulties and diagnosed mental health conditions among children and youth at a national level. This study, with the objective of examining the associations between meeting the recreational screen time recommendation and various indicators of mental health among children and youth in Canada, will help address this evidence gap, especially for younger children.

## Methods

***Data source***

This study utilized data from the 2019 Canadian Health Survey on Children and Youth (CHSCY), a cross-sectional survey conducted by Statistics Canada. The CHSCY covers a representative sample of children and youth aged 1 to 17 years living in the 10 provinces and the three territories. Excluded from the survey coverage were children and youth living on First Nations reserves and in other Indigenous settlements in the provinces and in foster homes and institutions. The survey sampling frame is based on the Canada Child Benefit File which covers 98% of the population in the provinces and 96% in the territories.^17^ The CHSCY is conducted under the authority of the *Statistics Act*, and therefore the use of these data for research purposes does not require research ethics board review.

This study included two age groups: children aged 5 to 11 years and youth aged 12 to 17 years. Children aged 3 to 4 years were excluded due to small sample sizes. For children and youth aged 5 to 17 years, a questionnaire was administered to the “person most knowledgeable” (PMK), who was most often a parent. A separate questionnaire was administered directly to youth; this questionnaire contained some of the same questions asked of children’s PMKs.

Surveys were mostly completed online; those who did not complete the online questionnaire by the deadline were contacted by a Statistics Canada interviewer to complete the questionnaire by telephone. Response rates were 57.8% for children and 41.3% for youth. Statistics Canada addressed total nonresponse by using a multistage process of adjusting the weight of the persons who responded to the survey to account for those who did not respond, to reduce any potential nonresponse bias.^17^

Of the 27771 CHSCY respondents, 26986 (97.2%) had complete sociodemographic data and were included in the current study. Respondents with missing data for specific mental health indicators were excluded from the respective analyses. There were no significant differences in sociodemographic characteristics of nonrespondents versus the study sample.

***Recreational screen time independent variable***

Respondents were asked how much time the child (5–11 years) or they (youth aged 12–17 years) spent using any electronic device such as a mobile device, computer, tablet, video game console or television while sitting down in the past 7 days. Response options were “no time,” “less than 3 hours,” “3 hours to less than 7 hours,” “7 hours to less than 14 hours,” “14 hours to less than 21 hours” or “21 hours or more.” Children and youth were classified as meeting the recommendation if they accumulated less than 14 hours per week of recreational screen time, which corresponds to the Canadian 24-H Movement Guidelines of2 hours or less per day.^3^ The response options were converted from hours per week to hours per day.

***Positive mental health outcomes***

Positive mental health outcomes were based on how they are defined and measured in the youth Positive Mental Health Surveillance Indicator Framework.^18^

**Perceived or self-rated mental health**

PMKs reported their perception of their child’s mental health, while youth self-rated their mental health. The five-point response scale options were dichotomized as “excellent or very good mental health” (versus “good,” “fair” or “poor”).^18^

**Life satisfaction**

Youth reported their general life satisfaction on a scale from 0 (“very dissatisfied”) to 10 (“very satisfied”). Responses of 9 or 10 were classified as high life satisfaction.^18^

**Happiness**

Youth who described themselves as “happy and interested in life” (versus “somewhat happy,” “somewhat unhappy,” “unhappy with little interest in life” or “so unhappy that life is not worthwhile”) were categorized as having high happiness.^18^

***Mental ill-health***

**Perceived stress**

Youth who reported that most of their days were “extremely” or “quite a bit stressful” (versus “not at all stressful,” “not very stressful” or “a bit stressful”) were categorized as often feeling stressed.^19^

**Appearing anxious or sad**

PMKs reported how frequently they thought the child or youth in their care appeared anxious, nervous or worried and how frequently they appeared sad or depressed. Participants whose PMK responded “daily” or “weekly” (versus “monthly,” “a few times a year” or “never”) were classified as often appearing anxious, nervous or worried or as often appearing sad or depressed.^20^

***Psychosocial difficulties***

PMKs reported whether the child or youth had difficulties with (1) remembering things; (2) concentrating on activities they enjoy doing; (3) accepting changes to their routine; (4) controlling their behaviour; and (5) making friends. Responses of “some difficulty,” “a lot of difficulty” or “cannot do at all” for any of these behaviours were classified as having at least some psychosocial difficulties (versus “no difficulty”).

These prompts are based on the 2016 Washington Group/UNICEF Child Functioning Module, which was designed to provide an estimate of the proportion of children with functional difficulties and was intended for use on national surveys.^20^ Although there are several components of this module, we only included those that were related to psychosocial difficulties.

***Diagnosed mental disorders and mental health service use***

**Diagnosed mood, anxiety or attention deficit hyperactivity disorder**

PMKs identified (by responding “yes” or “no”) whether the child or youth was ever diagnosed with an anxiety disorder, a mood disorder or attention deficit hyperactivity disorder (ADHD). The CHSCY did not include validated screening or diagnostic tools for diagnosing children or youth with these mental disorders; rather, PMKs reported whether the child or youth had ever been diagnosed with any one of these three disorders by a medical professional. Each of these disorders were analyzed as separate indicators.

**Required or received mental health services**

PMKs reported (by responding “yes” or “no”) whether the child or youth received care, in the past 12 months, for difficulties focusing or controlling behaviour; for mental health issues; from a psychologist or counsellor; or from a psychiatrist. Participants were categorized as having required or received mental health services if their PMK responded “yes” to any of these four questions.

***Covariates***

The following potential covariates were identified a priori: age (in years); identifying as Indigenous or as belonging to a racialized group (Arab, Black, Chinese, Filipino, Japanese, Korean, Latin American, South Asian, Southeast Asian, West Asian or Other versus White); immigrant status (landed immigrant, permanent resident or naturalized immigrant versus Canadian born); urban versus rural dwelling; household income adequacy (in quintiles); PMK’s self-rated mental health (excellent or very good versus good, fair or poor); and PMK’s self-reported stress (extremely or quite a bit stressful versus a bit stressful, not very stressful or not at all stressful).^21^-^24^

***Statistical analysis***

Proportions and 99% confidence intervals (CIs) were calculated for recreational screen time and mental health indicators, by age group and sex. We used 99% CIs, rather than 95% CIs, to account for possible spurious associations that can result when examining the relationship between one independent variable and multiple outcomes. Reporting by gender (specifically nonbinary) was not possible due to the small sample sizes. In addition, because the question on gender was PMK-reported for children and self-reported for youth, we chose to report on sex to avoid potential discrepancies between youth self-reports and PMK reports.

Multivariate logistic regression models were fitted to examine the relationship between meeting the recreational screen time recommendation and mental health indicators separately for children and youth. A secondary analysis assessed the association between amounts of daily recreational screen time and mental health indicators to explore dose–response relationships. Results are presented as adjusted odds ratios (aORs) with 99% CIs. Statistically significant results were identified for *p* values less than 0.01 and where CIs of odds ratios excluded the null (aOR = 1.0).

Estimates were weighted using sampling weights provided by Statistics Canada to account for survey design and nonresponse. Bootstrap weights were used for variance estimation. Analyses were conducted using SAS Enterprise Guide version 7.1 (SAS Institute Inc., Cary, NC, USA).

## Results

The majority of children (83.2%) and youth (56.9%) accumulated an average of less than 2 hours per day of leisure screen time (Table 1).

**Table 1:** Descriptive statistics for screen time variables, mental health outcomes and covariates for children and youth, 5–17 years, Canada, 2019

Variable	Children (5–11 years)			Youth (12–17 years)		
	Total (n = 16 272)	Females (n = 7886)	Males (n = 8386)	Total (n = 10 714)	Females (n = 5434)	Males (n = 5280)
Recreational screen time, %						
Meeting the recommendation^a^^b^	83.2	85.0	81.3	56.9	60.9	53.2
Amount of screen time per day^a^^b^ %						
No time	4.5	4.7	4.3	1.0	0.9^E^	1.2
Less than 30 minutes	19.5	20.9	18.2	9.2	9.7	8.7
30 minutes to less than 1 hour	30.5	31.5	29.5	22.7	24.0	21.4
1 hour to less than 2 hours	28.7	28.0	29.4	24.0	26.3	21.9
2 hours to less than 3 hours	11.5	10.5	12.4	20.8	19.8	21.7
3 hours or more	5.4	4.5	6.3	22.3	19.3	25.2
Positive mental health, %						
Excellent or very good mental health^a^^b^	83.0	85.2	80.9	66.2	58.3	73.7
High happiness^b^	n/a	n/a	n/a	64.5	60.6	68.1
High life satisfaction^b^	n/a	n/a	n/a	45.1	41.1	48.8
Mental ill-health, %						
Often feels stressed^b^	n/a	n/a	n/a	20.4	27.6	13.5
Often appears anxious, nervous or worried^a^	17.4	16.3	18.4	18.9	23.4	14.7
Often appears sad or depressed^a^	6.1	5.3	6.8	7.1	8.8	5.4
Psychosocial difficulties, %						
At least some psychosocial difficulties^a^	51.3	46.1	56.2	42.9	41.3	44.4
Diagnosed mental disorders and required or received mental health services, %						
Diagnosed mood disorder^a^	0.6	0.3^E^	0.9^E^	3.9	5.4	2.4
Diagnosed anxiety disorder^a^	3.2	2.6	3.8	7.6	9.7	5.7
Diagnosed ADHD^a^	7.6	4.3	10.7	10.2	6.4	13.7
Required or received mental health services^a^	15.7	11.9	19.3	18.4	19.8	17.1
Covariates, %						
Urban dwelling	82.2	82.3	82.1	81.6	82.0	81.1
Belonging to a racialized group or identifying as Indigenous	33.2	32.3	33.9	33.3	33.8	32.9
Immigrant (not Canadian born)	8.6	8.4	8.9	14.6	15.4	13.8
Household income adequacy						
Q1 (lowest quintile)	21.2	21.8	20.6	18.3	19.3	17.3
Q2	20.2	20.2	20.3	18.5	18.0	19.1
Q3	19.8	19.6	20.0	20.6	20.3	20.8
Q4	19.2	19.2	19.2	19.8	19.8	19.8
Q5 (highest quintile)	19.6	19.3	19.9	22.8	22.6	22.9
High PMK self-rated stress^a^	28.4	28.6	28.2	28.7	29.4	28.1
Excellent or very good PMK self-rated mental health^a^	72.7	72.8	72.6	70.8	71.0	70.7

**Source: **Canadian Health Survey on Children and Youth, 2019. 

**Abbreviations:** ADHD, attention deficit hyperactivity disorder; n/a, not available; PMK, person most knowledgeable.

^a^ Reported by the PMK.

^b^ Reported by youth. 

^E^ Interpret with caution due to high sampling variability. 

Most PMKs described their child as having “excellent or very good” mental health (83.0%); however, only 66.2% of youth self-rated their mental health as “excellent or very good.” About two-thirds of youth reported high happiness (64.5%); less than half reported high life satisfaction (45.1%); and less than a quarter reported often feeling stressed (20.4%) (Table 1).

PMKs reported that 17.4% of children and 18.9% of youth often appeared anxious, nervous or worried and that 6.1% of children and 7.1% of youth often appeared sad or depressed (Table 1).

PMKs reported psychosocial difficulties for 51.3% of children and 42.9% of youth. The prevalence of PMKs reporting that children and youth had been diagnosed with a mood disorder (0.6% and 3.9%), an anxiety disorder (3.2% and 7.6%) or ADHD (7.6% and 10.2%) or required or received mental health services (15.7% and 18.4%) was relatively low.

***Associations between meeting the recreational screen time recommendation and indicators of mental health among children (5–11 years)***

Compared with females who exceeded the recreational screen time recommendation, females who met the recommendation were less likely to have a PMK report that they often appeared sad or depressed (aOR=0.60; 99% CI: 0.37–0.99) or that they had been diagnosed with an anxiety disorder (aOR=0.49; 99% CI: 0.25–0.96) (Table 2).

**Table 2:** Association between adherence to the recreational screen time recommendation and indicators of mental health, children (5–11 years), Canada, 2019

Indicator	aOR (99% CI)		
	Total	Females	Males
Positive mental health			
Excellent or very good mental health	1.14 (0.92–1.43)	1.18 (0.84–1.65)	1.13 (0.84–1.52)
Mental ill-health			
Often appears anxious, nervous or worried	0.85 (0.70–1.04)	0.80 (0.60–1.07)	0.88 (0.67–1.17)
Often appears sad or depressed	0.81 (0.58–1.12)	0.60 (0.37–0.99)^*^	1.00 (0.66–1.51)
Psychosocial difficulties			
At least some psychosocial difficulties	0.84 (0.71–0.99)^*^	0.87 (0.68–1.12)	0.81 (0.65–1.02)
Diagnosed mental disorders and required or received mental health services			
Diagnosed mood disorder	0.81 (0.34–1.92)	0.35 (0.07–1.74)^E^	1.07 (0.38–3.06)^E^
Diagnosed anxiety disorder	0.79 (0.51–1.22)	0.49 (0.25–0.96)^*^	1.07 (0.61–1.91)
Diagnosed ADHD	0.86 (0.64–1.14)	0.63 (0.37–1.07)	0.96 (0.69–1.34)
Required or received mental health services	0.83 (0.67–1.03)	0.80 (0.56–1.14)	0.85 (0.65–1.11)

Source: Canadian Health Survey on Children and Youth, 2019. 

**Abbreviations: **ADHD, attention deficit hyperactivity disorder; aOR, adjusted odds ratio; CI, confidence interval; PMK, person most knowledgeable.

**Notes: **Models have been adjusted for age, identifying as Indigenous or as belonging to a racialized group, immigrant status, urban or rural residence, household income adequacy, PMKreported mental health and PMK-reported stress. The reference group exceeded the recreational screen time recommendation of < 2 hours/day. 

^a^ Reported by the PMK. 

^E^ Interpret with caution due to high sampling variability. 

*p < 0.01.

***Associations between meeting the recreational screen time recommendation and indicators of mental health among youth (12–17 years)***

Among both female and male youth, meeting the recreational screen time recommendation was associated with a greater likelihood of reporting high happiness (aORs: 1.84 and 1.74) and high life satisfaction (aORs: 1.80 and 1.64) and a lower likelihood of often feeling stressed (aORs: 0.64 and 0.74) (Table 3). In addition, meeting the recommendation was associated with a greater likelihood of female youth reporting “excellent or very good” mental health (aOR = 1.65; 99% CI: 1.33–2.04) and a lower likelihood of often appearing anxious, nervous or worried (aOR= 0.77; 99% CI: 0.60–0.97), often appearing sad or depressed (aOR=0.68; 99% CI: 0.49–0.94) and being diagnosed with an anxiety disorder (aOR=0.65; 99% CI: 0.46–0.92). Among male youth, meeting the recommendation was associated with a lower likelihood of experiencing at least some psychosocial difficulties (aOR=0.79; 99% CI: 0.64–0.97).

**Table 3:** Association between adherence to the recreational screen time recommendation and indicators of mental health, youth aged 12–17 years, Canada, 2019

Indicator	aOR (99% CI)		
	Total	Females	Males
Positive mental health			
Excellent or very good mental health^a^	1.44 (1.23–1.68)^*^	1.65 (1.33–2.04)^*^	1.25 (0.99–1.57)
High happiness^a^	1.80 (1.54–2.09)^*^	1.84 (1.48–2.29)^*^	1.74 (1.40–2.15)^*^
High life satisfaction^a^	1.71 (1.48–1.98)^*^	1.80 (1.44–2.23)^*^	1.64 (1.34–2.01)^*^
Mental ill-health			
Often feels stressed^a^	0.68 (0.56–0.82)^*^	0.64 (0.50–0.82)^*^	0.74 (0.56–0.99)^*^
Often appears anxious, nervous or worried^b^	0.85 (0.71–1.03)	0.77 (0.60–0.97)^*^	0.98 (0.75–1.30)
Often appears sad or depressed^b^	0.76 (0.59–0.99)^*^	0.68 (0.49–0.94)^*^	0.89 (0.58–1.37)
Psychosocial difficulties			
At least some psychosocial difficulties^b^	0.81 (0.70–0.94)^*^	0.84 (0.68–1.04)	0.79 (0.64–0.97)^*^
Diagnosed mental disorders and mental health services^b^			
Diagnosed mood disorder	0.79 (0.55–1.15)	0.74 (0.48–1.12)	0.90 (0.48–1.71)
Diagnosed anxiety disorder	0.69 (0.53–0.90)^*^	0.65 (0.46–0.92)^*^	0.75 (0.49–1.13)
Diagnosed ADHD	0.86 (0.67–1.12)	0.90 (0.56–1.45)	0.85 (0.62–1.15)
Required or received mental health services	0.88 (0.72–1.06)	0.82 (0.62–1.06)	0.95 (0.72–1.25)

**Source:** Canadian Health Survey on Children and Youth, 2019.

**Abbreviations: **ADHD, attention deficit hyperactivity disorder; aOR, adjusted odds ratio; CI, confidence interval; PMK, person most knowledgeable. 

**Notes:** Models have been adjusted for age, identifying as Indigenous or as belonging to a racialized group, immigrant status, urban or rural residence, household income adequacy, PMKreported mental health and PMK-reported stress. The reference group did not meet the recreational screen time recommendation of < 2 hours/day.

^a^ Reported by youth. 

^b^ Reported by the PMK. 

*p < 0.01. 

***Dose–response associations between recreational screen time and indicators of mental health***

Compared to female children who accumulated less than 1 hour per day of recreational screen time, those who exceeded the recommendation of 2 hours per day had a greater likelihood of being diagnosed with an anxiety disorder (aOR = 2.08; 99% CI: 1.02–4.28); no differences were observed between those with less than 1hour per day and 1 to less than 2 hours per day of screen time (Table 4). Among male children, 2 hours or more per day of screen time was associated with a greater likelihood of experiencing psychosocial difficulties (aOR = 1.29; 99% CI: 1.02–1.63). Engaging in 1 to less than 2 hours per day of recreational screen time was associated with a greater likelihood of male children requiring or receiving mental health services (aOR=1.31; 99% CI: 1.01–1.70).

**Table 4:** Odds ratios for mental health by amounts of daily recreational screen time, children (5–11 years), Canada, 2019

Indicator	Total							Females							Males						
	< 1 h	1 to < 2 h			≥ 2 h			< 1 h	1 to < 2 h			≥ 2 h			< 1 h	1 to < 2 h			≥ 2 h		
	Ref.	aOR	99% LCL	99% UCL	aOR	99% LCL	99% UCL	Ref.	aOR	99% LCL	99% UCL	aOR	99% LCL	99% UCL	Ref.	aOR	99% LCL	99% UCL	aOR	99% LCL	99% UCL
Positive mental health^a^																					
Excellent or very good mental health	Ref.	0.86	0.7	1.07	0.83	0.65	1.05	Ref.	0.93	0.68	1.28	0.83	0.58	1.19	Ref.	0.81	0.61	1.09	0.81	0.59	1.11
Mental ill-health^a^																					
Often appears anxious, nervous or worried	Ref.	1.08	0.9	1.31	1.21	0.98	1.49	Ref.	1.02	0.78	1.35	1.26	0.93	1.72	Ref.	1.13	0.87	1.47	1.19	0.89	1.59
Often appears sad or depressed	Ref.	0.99	0.75	1.31	1.23	0.87	1.74	Ref.	0.86	0.55	1.35	1.58	0.93	2.68	Ref.	1.07	0.73	1.56	1.03	0.67	1.58
Psychosocial difficulties^a^																					
At least some psychosocial difficulties	Ref.	1.09	0.95	1.26	1.23^*^	1.03	1.46	Ref.	1.05	0.86	1.29	1.17	0.90	1.52	Ref.	1.13	0.92	1.37	1.29^*^	1.02	1.63
Diagnosed mental disorders and mental health services^a^																					
Diagnosed mood disorder	Ref.	1.64^E^	0.66	4.08	1.56^E^	0.61	3.98	Ref.	^F^	^F^	^F^	^F^	^F^	^F^	Ref.	1.54^E^	0.53	4.43	1.17^E^	0.38	3.49
Diagnosed anxiety disorder	Ref.	1.12	0.75	1.66	1.33	0.84	2.12	Ref.	1.04^E^	0.55	1.98	2.08^*^^E^	1.02	4.28	Ref.	1.12	0.66	1.88	0.98	0.53	1.81
Diagnosed ADHD	Ref.	1.17	0.88	1.56	1.25	0.92	1.70	Ref.	0.96	0.55	1.68	1.56	0.88	2.77	Ref.	1.26	0.90	1.76	1.15	0.80	1.66
Required or received mental health services	Ref.	1.22^*^	1.00	1.49	1.30^*^	1.03	1.64	Ref.	1.10	0.80	1.52	1.30	0.88	1.91	Ref.	1.31^*^	1.01	1.70	1.31	0.98	1.75

**Source:** Canadian Health Survey on Children and Youth, 2019.

**Abbreviations: **ADHD, attention deficit hyperactivity disorder; aOR, adjusted odds ratio; h, hour; LCL, lower confidence limit; PMK, person most knowledgeable; Ref., reference; UCL, upper confidence limit. 

**Notes: **Models have been adjusted for age, identifying as Indigenous or as belonging to a racialized group, immigrant status, urban or rural residence, household income adequacy, PMK-reported mental health and PMK-reported stress. The reference group had < 1 hour/day of screen time. 

^a^ Reported by the PMK. 

^E^ Interpret with caution due to high sampling variability. 

^F^ Too unreliable to be published due to high sampling variability. 

* *p* < 0.01. 

A dose–response relationship was observed among female youth. As recreational screen time amounts increased, the likelihood of often feeling stressed, often appearing anxious, nervous or worried, experiencing psychosocial difficulties, and requiring or receiving mental health services also increased, whereas the likelihood of reporting high levels of positive mental health indicators decreased (Table 5).

**Table 5:** Odds ratios for mental health by hours of daily recreational screen time, youth 12–17 years, Canada, 2019

Indicator	Total										Females										Males									
	< 1 h	1 to < 2 h			2 to < 3 h			≥ 3 h			< 1 h	1 to < 2 h			2 to < 3 h			≥ 3 h			< 1 h	1 to < 2 h			2 to < 3 h			≥ 3 h		
	Ref.	aOR	99% LCL	99% UCL	aOR	99% LCL	99% UCL	aOR	99% LCL	99% UCL	Ref.	aOR	99% LCL	99% UCL	aOR	99% LCL	99% UCL	aOR	99% LCL	99% UCL	Ref.	aOR	99% LCL	99% UCL	aOR	99% LCL	99% UCL	aOR	99% LCL	99% UCL
Positive mental health^a^																														
Excellent or very good mental health	Ref.	0.81^*^	0.66	0.99	0.70^*^	0.57	0.88	0.57^*^	0.46	0.71	Ref.	0.76	0.57	1.00	0.61^*^	0.46	0.82	0.47^*^	0.34	0.63	Ref.	0.89	0.65	1.21	0.84	0.60	1.16	0.71^*^	0.53	0.95
High happiness	Ref.	0.82	0.67	1.00	0.60^*^	0.48	0.75	0.44^*^	0.36	0.54	Ref.	0.66^*^	0.50	0.87	0.55^*^	0.40	0.74	0.37^*^	0.27	0.50	Ref.	1.06	0.78	1.43	0.68^*^	0.50	0.92	0.53^*^	0.40	0.70
High life satisfaction	Ref.	0.75^*^	0.62	0.91	0.59^*^	0.48	0.72	0.45^*^	0.37	0.56	Ref.	0.68^*^	0.52	0.90	0.60^*^	0.45	0.81	0.36^*^	0.26	0.50	Ref.	0.82	0.62	1.08	0.59^*^	0.45	0.78	0.54^*^	0.41	0.71
Mental ill-health																														
Often feels stressed^a^	Ref.	1.19	0.93	1.51	1.41^*^	1.09	1.82	1.78^*^	1.38	2.29	Ref.	1.24	0.91	1.68	1.42^*^	1.02	1.99	2.10^*^	1.51	2.92	Ref.	1.06	0.70	1.61	1.34	0.90	1.99	1.42	0.97	2.07
Often appears anxious, nervous or worried^b^	Ref.	0.94	0.74	1.19	1.04	0.81	1.33	1.24	0.96	1.59	Ref.	1.20	0.88	1.65	1.31	0.94	1.81	1.54^*^	1.10	2.17	Ref.	0.62^*^	0.41	0.92	0.74	0.50	1.09	0.94	0.66	1.34
Often appears sad or depressed^b^	Ref.	0.99	0.69	1.41	1.23	0.83	1.80	1.37	0.96	1.96	Ref.	1.17	0.73	1.86	1.50	0.92	2.45	1.67^*^	1.06	2.63	Ref.	0.72	0.42	1.25	0.91E	0.48	1.73	1.06	0.61	1.85
Psychosocial difficulties^b^																														
At least some psychosocial difficulties	Ref.	1.08	0.90	1.30	1.17	0.96	1.44	1.38^*^	1.13	1.68	Ref.	1.21	0.93	1.58	1.23	0.92	1.64	1.38^*^	1.03	1.84	Ref.	0.95	0.73	1.24	1.11	0.84	1.48	1.36^*^	1.03	1.79
Diagnosed mental disorders and mental health services^b^																														
Diagnosed mood disorder	Ref.	1.23	0.74	2.04	1.45	0.86	2.45	1.33	0.80	2.22	Ref.	1.50	0.81	2.77	1.71	0.93	3.15	1.63	0.88	3.02	Ref.	0.70^E^	0.25	1.93	1.04^E^	0.41	2.68	0.93^E^	0.41	2.09
Diagnosed anxiety disorder	Ref.	1.18	0.83	1.67	1.43	0.99	2.06	1.69^*^	1.17	2.44	Ref.	1.38	0.87	2.17	1.83^*^	1.15	2.91	1.77^*^	1.08	2.88	Ref.	0.87	0.49	1.53	0.95	0.52	1.74	1.53	0.89	2.61
Diagnosed ADHD	Ref.	1.31	0.92	1.87	1.06	0.73	1.55	1.53^*^	1.07	2.20	Ref.	1.46	0.80	2.67	1.00^E^	0.52	1.91	1.68	0.87	3.26	Ref.	1.22	0.79	1.89	1.08	0.69	1.71	1.47	0.98	2.21
Required or received mental health services	Ref.	1.10	0.85	1.42	1.06	0.81	1.39	1.32^*^	1.01	1.72	Ref.	1.51^*^	1.08	2.11	1.37	0.96	1.95	1.62^*^	1.12	2.35	Ref.	0.75	0.51	1.10	0.81	0.54	1.20	1.06	0.73	1.54

**Source: **Canadian Health Survey on Children and Youth, 2019.

**Abbreviations: **ADHD, attention deficit hyperactivity disorder; aOR, adjusted odds ratio; LCL, lower confidence limit; PMK, person most knowledgeable; Ref., reference; UCL, upper confidence limit. 

**Notes:** Models have been adjusted for age, identifying as Indigenous or as belonging to a racialized group, immigrant status, urban or rural residence, household income adequacy, PMK-reported mental health and PMK-reported stress. The reference group has < 1 hour/day of screen time. 

^a^ Reported by youth. 

^b^ Reported by the PMK. 

^E^ Use with caution due to high sampling variability. 

* *p* < 0.01. 

For male youth, increasing screen time to 2 hours or more per day was associated with lower odds of reporting high levels of positive mental health indicators (Table5). Having higher odds of experiencing psychosocial difficulties was only associated with recreational screen time amounts of more than 3 hours per day. Conversely, males accumulating less than the recommended amount of screen time per day (1to < 2 hours) had a lower likelihood of appearing anxious, nervous or worried.

## Discussion

In this study we examined the relationship between meeting the 24-H Movement Guidelines’ recreational screen time recommendation and various indicators of mental health among children and youth in Canada. We found that adhering to the recommendation was positively associated with positive mental health indicators and negatively associated with indicators of mental ill-health, psychosocial difficulties and diagnosed mental health conditions, with differences in effect size across sex and age groups.

***Positive mental health***

Our findings suggest that meeting the recreational screen time recommendation was associated with all examined indicators of positive mental health among female youth and with happiness and life satisfaction among male youth. A population-based Canadian study found a similar, albeit inverted, significant association, whereby exceeding 2 hours per day of screen time was associated with worse self-rated mental health (i.e. good, fair or poor self-rated mental health) among youth.^25^

We observed a dose–response relationship between daily screen time amounts and positive mental health among male and female youth. Studies of North American and European youth have also found similar dose–response relationships between screen time amounts, happiness and life satisfaction.^26^,^27^ Twenge et al.^27^ found a U-shaped relationship between screen time and unhappiness, with the lowest prevalence of unhappiness reported when using electronic devices between <1hour and 1–2 hours per week, with variations by school grade and device type. Among female youth, life satisfaction decreased after 1 hour per day of screen time, whereas among male youth the decrease occurred after 1.5 hours per day of screen time.^26^ This aligns with our findings of lower odds of reporting high happiness and life satisfaction with increasing amounts of screen time. Other studies, however, have found no association between recreational screen time and indicators of positive mental health.^13^,^28^ These null associations may be due to differences in screen time and mental health measures, compared to our study, as well as differing population coverage, year of data collection, covariates, and PMK- versus child- or youth-reported data. We did not find a significant relationship between meeting the recreational screen time recommendation and PMK-rated child mental health. Interpreting the absence of significant associations is challenging as very few studies have examined this association in children.

***Mental ill-health***

We found that meeting the recreational screen time recommendation was associated with a lower likelihood of female children and youth appearing sad or depressed and a lower likelihood of female youth appearing anxious, nervous or worried. The measures of mental ill-health we used in our study rely on questions about usual feelings of anxiety, nervousness or worry as well as sadness or depression, rather than symptoms of anxiety or depression, as commonly reported in the literature.^14^,^15^,^29^-^32^ Although symptoms of anxiety or depression may be a proxy to the measures we used, they are not necessarily directly comparable. Some previous studies found positive associations between screen time and symptoms of anxiety or depression while others found none.^29^-^31^ Studies examining gender differences found that female youth who played video games or watched TV for more than 3hours per day had more symptoms of anxiety and depression than those with less screen time; however, the opposite or no association was found among male youth.^29^,^31^,^33^ One study suggests that media use may be a protective factor for male youth, as those who spend more time playing video games and watching TV report fewer symptoms of anxiety and depression.^33^ Our findings also show no associations between screen time and male youth appearing anxious, nervous or worried or appearing sad or depressed. The types of devices that male and female youth predominantly use could explain this difference^33^-^37^ as screen types have varying associations with mental health.^14^,^38^ For example, social media use can foster social comparison, which can negatively affect mental health, but such comparisons are less common when playing video games or watching TV.^38^ However, limited research is available to examine the association between types of screen-based activities and mental health, especially among younger children.

Our findings also suggest that female and male youth who met the recommendation were less likely to often feel stressed. A study of adolescents from across 38 countries in Europe and North America found a positive linear association between the amount of screen time and levels of school-related stress with no apparent gender differences,^39^ whereas a study of Ontario adolescents found no significant association.^40^ Overall, our findings suggest that adherence to the screen time recommendation is associated with lower likelihoods of indicators of mental ill-health, especially among female youth.

***Psychosocial difficulties***

Male youth who met the screen time recommendation were less likely to experience psychosocial difficulties, but we found no significant association with children or female youth. The literature examining associations between screen time and psychosocial difficulties is inconsistent. Large studies of children and youth from Australia and the United States found linear and U-shaped relationships between screen time and different psychosocial difficulties.^41^,^42^

The data we used in our study came from asking PMKs to report on their young or adolescent child’s psychosocial difficulties, which may have led to discrepancies in the perceived difficulties. One study found that parents of boys reported more psychosocial difficulties than did the parents of girls, and that children tended to report more symptoms than their parents.^43^

***Diagnosed mental disorders and mental health services***

We found that female children and youth who met the screen time recommendation were less likely to be diagnosed with an anxiety disorder. In addition, female youth who accumulated 1 to less than 2 hours or 3 or more hours per day of recreational screen time were more likely to require or receive mental health services than those with less than 1 hour of daily screen time. Previous research suggests that youth who used screens for 4 to 7 hours per day were more likely to be diagnosed with depression or anxiety and seek mental health care than their peers who used screens for 1 hour per day.^32^,^44^ Poor mental health literacy, lack of emotional competence and fewer intimate relationships have been identified as barriers in seeking mental health care, particularly among male youth.^45^,^46^ This may explain in part why we only observed the association between screen time and mental health service use among female youth in our study.

Overall, we found several significant associations between adherence to the recreational screen time recommendation and indicators of mental health among youth, with notable sex differences. However, very few statistically significant associations were found among children, which may be due to insufficient power to detect associations. While the available research shows that accumulating excess screen time in early childhood is associated with mental ill-health outcomes, it is possible that these outcomes may not be as apparent until adolescence.^11^,^47^-^49^ In addition, the literature suggests that certain screen-based devices are more harmful to mental health compared to others, and the types of devices that children and youth predominantly use are different.^7^,^38^

***Strengths and limitations***

Strengths of this study include the representative sample, and the comprehensive range of mental health indicators examined. In addition, in recognition of sex differences in screen time and mental health indicators, we explored sex-specific associations. Lastly, our study includes children as young as 5 years, which helps address the evidence gap in the literature for this population group.

However, this study does have several limitations. This work does not encompass the full spectrum of mental disorders and symptoms because data were not collected or sample sizes were too small to report. The survey question about recreational screen time included categorical response options across a 7-day period, which did not directly align with the recommended threshold of 2 hours per day or less. In addition, some of the survey questions used to assess the mental health outcomes in this study are not from validated mental health scales. The data were also collected retrospectively and were primarily based on self-report and report by the PMK, and may therefore be prone to recall, social desirability and informant biases.^43^,^50^,^51^ Lastly, the CHSCY’s cross-sectional design prevents inferences on causality and directionality. There is some evidence to suggest a bidirectional relationship; while screen time may be a predictor of mental health, pre-existing mental health problems or stressors may also predict screen time use.^48^,^52^

***Future research and public health implications***

Other than this present study, there has been no research examining the associations between recreational screen time and mental health of children and youth by gender or sex; future studies examining gender and sex are needed to validate our findings. Given the cross-sectional nature of the present study and the potential for a bidirectional relationship, future longitudinal studies are needed to confirm the direction of effect. In addition, future work is needed to explore the association between recreational screen time and positive mental health among younger children, and the types of screens and programs being used by children and youth.

Understanding the dose–response of recreational screen time associations with the mental health among children and youth is important for public health intervention design. Previous work that supported the development of the 24-H Movement Guidelines suggested that engaging in recreational screen time for more than 2 hours per day is associated with a multitude of health problems.^2^,^5^ Most previous research had been among youth, with limited evidence among younger children. Our findings support the 2-hour-per-day limit, but also suggest that in some cases, shorter amounts of screen time are associated with lower life satisfaction and happiness and greater anxiety, and higher doses are associated with poorer mental health. Promotion of the current limit of 2 hours per day remains an important intervention.

## Conclusion

Female children who meet the 24-H Movement Guidelines’ screen time recommendation are less likely to appear sad or depressed and be diagnosed with an anxiety disorder. Youth who meet the screen time recommendation may have better mental health than those exceed the recommendation. Findings also suggest a dose–response relationship, where higher screen time amounts are associated with a reduced likelihood of reporting high levels of positive mental health indicators, among youth. As screen-based devices continue to be a part of everyday life for children and youth, it is important to monitor how their use affects both their mental and physical health, and to encourage healthy screen time habits. Future work is needed to examine if the association between recreational screen time and mental health has changed as a result of the COVID-19 pandemic and to explore longitudinal trends and associations.

## Acknowledgements

We would like to thank those at Statistics Canada who designed the 2019 Canadian Health Survey on Children and Youth (CHSCY) and those who collected and processed the data.

## Funding

None.

## Conflicts of interest

The authors have no conflicts of interests to disclose.

## Authors’ contributions and statement

ST – Conceptualization, methodology, formal analysis, writing – original draft.

CW – Conceptualization, methodology, writing – review & editing.

SPW – Conceptualization, methodology, writing – review & editing.

MV – Conceptualization, methodology, writing – review & editing.

KCR – Conceptualization, methodology, writing – review & editing.

MTB – Conceptualization, methodology, writing – review & editing.

All authors have reviewed and approved the final manuscript.

The content and views expressed in this article are those of the authors and do not necessarily reflect those of the Government of Canada.
